# Cost-Effectiveness of Angle-Based Minimally Invasive Glaucoma Surgery (MIGS): A Systematic Review of Economic Evaluations

**DOI:** 10.3390/medsci14060664

**Published:** 2026-10-12

**Authors:** See Teng Tan, Shayne S. Tan, Hla M. Htoon, Shamira A. Perera, Tina T. Wong, Ryan E. K. Man

**Affiliations:** 1 Singapore National Eye Centre, Singapore 168751, Singapore; seeteng.tan@mohh.com.sg (S.T.T.); shamira.perera@singhealth.com.sg (S.A.P.); tina.wong.tl@singhealth.com.sg (T.T.W.); 2 Singapore Eye Research Institute (SERI), The Academia, 20 College Road Discovery Tower Level 6, Singapore 169856, Singapore; gmsv2552@visitor.nus.edu.sg; 3 Ophthalmology and Visual Sciences Academic Clinical Programme, Duke-NUS Medical School, Singapore 169857, Singapore

**Keywords:** minimally invasive glaucoma surgery, cost-effectiveness, systematic review

## Abstract

**Background/Objectives:** The upfront costs of angle-based minimally invasive glaucoma surgery (MIGS) maybe offset by reduced medication use and subsequent interventions. This systematic review synthesizes available cost-effectiveness analyses of angle-based MIGS in adults with primary open-angle glaucoma (POAG). **Methods:** A systematic review of PubMed, Embase, and Scopus databases from inception to 18 March 2026 for full-text, English-language economic evaluations reporting incremental cost-effectiveness/utility ratios (ICER/ICUR) or quality-adjusted life years (QALYs). Methodological quality was assessed using the Drummond checklist and, reporting completeness was assessed using CHEERS 2022. Findings were synthesized descriptively by intervention and comparator group, distinguishing standalone procedures from those combined with cataract surgery. No certainty assessment was undertaken. **Results:** Sixteen model-based economic evaluations encompassing 39 comparisons were included, with time horizons ranging between 1-year to lifetime. Drummond scores ranged from 8.0–9.5/10. Reported findings generally favored angle-based MIGS compared with medical therapy or cataract surgery (CS) alone, with 61.5% of comparisons categorized as cost-effective and 33.3% as economically dominant. The remaining 5.1% (*n* = 2) evaluations were not cost-effective at the stated willingness-to-pay thresholds. Of 19 evaluations derived from 10 studies, implant-based procedures combined with CS dominated CS alone across 7 evaluations and was cost-effective across 10 evaluations. Reporting of heterogeneity, distributional effects and stakeholder engagement was inconsistent. Evidence was limited by reliance on modelled outcomes, heterogenous assumptions and limited representation of lower-resource settings. **Conclusions:** Currently, angle-based MIGS procedures are cost-effective for the management of mild-moderate glaucoma as compared to medical therapy or standalone CS. Future research should prioritize head-to-head comparisons of different MIGS devices and focus on evaluating the cost-effectiveness of real-world implementation particularly in lower-resource settings. **Funding:** National Medical Research Council support for R.E.K.M. **Registration:** PROSPERO CRD420261462492.

## 1. Introduction

Glaucoma is an irreversible blinding disease characterized by progressive apoptosis of retinal ganglion cells, optic nerve atrophy, and visual field defects. Primary open-angle glaucoma (POAG) is the most common subtype, affecting 52.7 million individuals worldwide in 2020 [1]. Defined by glaucomatous optic neuropathy in the presence of an open drainage angle and elevated intraocular pressure, projections indicate that the global population of individuals aged ≥40 years with POAG will increase from 81 million in the year 2024 to 185 million by 2060. Additionally, an estimated 6 million early-onset POAG cases are anticipated among individuals aged 20 to 39 years due to high myopia [2].

IOP is currently the only known modifiable risk factor for POAG progression [3,4,5,6], yet first line treatment like topical drops and laser therapy [7], often fail to achieve adequate control due to high non-adherence rates or inadequate responses that put patients at risk for irreversible glaucomatous damage [8,9]. While traditional filtering surgeries such as trabeculectomy effectively lower IOP by up to 50% [10], they carry significant risks of severe complications including persistent hypotony, bleb leak, and endophthalmitis [11]. Minimally invasive glaucoma surgery (MIGS) has thus emerged as an attractive alternative treatment option for mild to moderate open angle glaucoma, offering a favourable safety profile via an ab interno approach at the expense of a more modest IOP lowering effect compared to filtering surgery [12,13]. In addition to its IOP-lowering efficacy, MIGS procedures have also been shown to significantly reduce medication burden, delay visual field progression, and decrease in post-operative visits [14,15,16].

MIGS procedures can be broadly classified into angle-based and non-angle-based procedures. Angle-based MIGS target the conventional physiological outflow system, including the trabecular meshwork (TM), Schlemm’s canal (SC), collector channels to lower IOP, and are often, but not always, performed in combination with cataract surgery (CS) [17,18]. These procedures reduce outflow resistance using micro-bypass stents (e.g., iStent [Glaukos Corp, California, USA] [19], Hydrus Microstent [Alcon Inc., Texas, USA]) [20], SC Visco dilation (e.g., ab interno canaloplasty, iTrack Advance [Nova Eye Medical Limited, South Australia, Australia], OMNI Surgical system [Sight Sciences Inc, California, USA]), or TM cleavage or resection (e.g., ab interno trabeculotomy and gonioscopy assisted transluminal trabeculotomy (GATT)). In contrast, non-angle-based MIGS bypass the conventional aqueous outflow pathway entirely by creating alternative pathways through which the aqueous is redirected [21,22]. While early data has shown that non-angle implant-based MIGS may be able to achieve good IOP-lowering efficacy, concerns over long-term safety have led to widespread recall of these devices [23].

Despite extensive research supporting the clinical efficacy of angle-based MIGS in the management of POAG, their real-world adoption is heavily influenced by economic factors, such as cost-effectiveness, reimbursement policies, and healthcare infrastructure [24]. From a healthcare-system and payer perspective, the high upfront costs of these devices and procedures raise significant economic concerns. For example, a 2019 study showed that the wholesale acquisition price was $2275 USD for a single iStent injector with 2 iStent implants, and USD $1900 for a Hydrus microstent [25]. In comparison, generic latanoprost costs USD $19.12 per bottle [26], rendering a 2-year supply roughly 4 times cheaper than the initial implantation cost of either a single implantation of the iStent or Hydrus microstents. While the pricing of these devices in different healthcare systems is complicated by variations in government subsidies, reimbursement policies, and private healthcare insurance coverage [27], financing these costly MIGS procedures must make economic sense to the respective stakeholders. Combined with their modest long-term IOP reduction [18,28], robust cost-effectiveness evaluations are therefore essential to justify their adoption into primary glaucoma care [29]. As such, elucidating the cost-effectiveness of angle-based MIGS procedures using metrics such as Quality-Adjusted Life Years (QALYs), Incremental Cost-Effectiveness Ratios (ICERs), and Incremental Cost-Utility Ratios (ICURs), is important in determining the place of MIGS in the POAG treatment paradigm, as they enable direct comparison with more established glaucoma intervention modalities, such as medications and filtering surgery.

This systematic review therefore aims to synthesize the available literature evaluating the cost-effectiveness of angle-based MIGS against both medication and surgical intervention modalities in the management of POAG, and to examine their implications for clinical decision-making, particularly, whether the higher upfront costs of MIGS are offset by reductions in medication use and the need for subsequent interventions.

## 2. Methodology

### 2.1. Reporting Guidelines and Registration

This systematic review was conducted in accordance with Preferred Reporting Items for Systematic Reviews and Meta-Analyses (PRISMA) 2020 guidelines [30]. The review was registered with PROSPERO (CRD 420261462492), with review method available within the PROSPERO record; no separate review protocol was prepared.

### 2.2. Ethics Statement

This review synthesized previously conducted studies and does not contain any studies with human participants or animals performed by any of the authors.

### 2.3. Search Strategy

Comprehensive electronic searches were performed from database inception until 18 March 2026 across MEDLINE/PubMed, EMBASE and Scopus, to identify published cost-effectiveness analyses evaluating MIGS interventions. Specific search terms were utilized for each of these databases. The search strategy combined terms related to glaucoma, MIGS and economic outcomes. The full search strategy is outlined in Supplementary Table S1.

### 2.4. Eligibility Criteria

We limited our search to English-language clinical studies involving human subjects, published from study inception up until 18 March 2026. Clinical trials and observational studies were not eligible solely on the basis of reporting clinical effectiveness outcomes; they were eligible only if they also reported an economic evaluation meeting the review criteria. The inclusion criteria were based on the Population, Intervention, Comparison, and Outcomes (PICO) [31], and included patients aged 18 years or older with a diagnosis of POAG who underwent primary angle-based MIGS procedure in at least one eye. These were assessed either as standalone procedures or combined with CS. The evaluated comparators consisted of standard of care topical medication regimens, selective laser trabeculoplasty (SLT), standalone CS, filtration surgery (trabeculectomy) or MIGS-versus-MIGS comparisons.

Eligible evaluations were required to report an incremental cost-effectiveness ratio (ICER) or incremental cost-utility ratio (ICUR). Conference abstract without an eligible full report, reviews, editorials and studies without incremental value metrics (ICER, ICUR) were excluded. For synthesis, studies and comparisons were grouped by intervention and comparator, with standalone procedures considered separately from procedures combined with CS.

### 2.5. Study Selection

Two reviewers (TST and SST) screened titles and abstracts independently, followed by full-text assessment of eligible studies. Reviewers were masked to each other’s decisions, and disagreements were resolved through discussion or third-party adjudication with a senior author (REKM).

### 2.6. Data Extraction

Data were extracted by one reviewer (TST) and independently verified by a second reviewer (SST) into a prespecified data extraction form that was developed to systematically capture the following: first author, year and country of publication; sample size and setting; time horizon; type of study or intervention; the outcomes addressed in the economic evaluation; discount rates; type of economic evaluation; key findings of the study and methodological insights. Missing or unclear information was recorded as not reported (NR), without imputation.

### 2.7. Outcomes and Economic Measures

Outcome measures specifically captured included ICER, ICUR and QALY gains analysis relative to the stated willingness-to-pay (WTP) thresholds. An ICER expressed the difference in costs between strategies divided by the difference in their health effects. An ICUR expresses incremental cost per QALY gained, per progression-free life-year gained; or per blind eye avoided, as reported by the original studies. Economic dominance was defined as lower costs and greater health benefits for the intervention relative to its comparator.

Results were reported according to the PRISMA guidelines for systematic reviews [30]. We initially considered conducting a meta-analysis to quantitatively synthesize findings across the included studies. However, this was not considered feasible because of substantial heterogeneity in study designs and methodologies, differences in stakeholder perspectives, and inconsistencies in currency valuation and study periods.

### 2.8. Data Preparation and Synthesis

A structured narrative synthesis was undertaken because differences in economic methods, perspective, currencies, price years and time horizons precluded meaningful quantitative pooling. Study characteristics, comparison-level economic findings and methodological appraisal results were tabulated. Economic findings were classified at the comparison level. Extracted intervention and comparator characteristics were used to assign each eligible comparison to the corresponding descriptive synthesis group. Comparisons were grouped by device-based or non-device-based angle-based MIGS and comparator type, with standalone procedures considered separately from those combined with cataract surgery. Studies reporting multiple eligible comparisons could contribute to more than one group. Comparisons showing lower costs and greater health benefits for the intervention classified as dominant. For interventions associated with higher cost and greater health benefits, comparisons were classified as cost-effective when the reported ICER or ICUR was at or below the appliable WTP threshold and as not cost-effective when it exceeded that threshold. Interventions associated with higher costs and poorer health outcomes were classified as dominated and included within the non-cost-effective category. Sensitivity analysis reported within the included economic evaluations were summarized descriptively. Economic results were retained in their original currencies, price years and outcome units; no inflation adjustment or currency conversion was undertaken.

### 2.9. Quality Assessment

The methodological quality of the included economic evaluations was independently assessed by two reviewers (TST an SST) using the 10-item Drummond checklist [32]. This checklist addresses: (1) the clarity of the research question; (2) the description of the study and interventions; (3) the study design; (4–6) the identification, measurement, and valuation of costs and consequences; (7) whether discounting was carried out; (8) incremental analysis; (9) presentation of results with uncertainty and sensitivity analyses; and (10) discussion of results in the context of policy relevance and existing literature. Each item was scored as 1 (fully met), 0.5 (partially met), or 0 (not met or not reported), yielding a maximum total score of 10. Methodological quality was categorized as Poor (1–3 points), Average (4–7 points), or Good (8–10 points) [33]. The detailed Drummond checklist scores are provided in Supplementary Chart S2.

Reporting completeness was assessed by using the CHEERS 2022 checklist [34]. The first reviewer performed the initial assessment, and a second reviewer independently verified all checklist entries. The updated CHEERS checklist encompasses 28 specific items, which are organized into 7 overarching categories: (i) title, (ii) abstract, (iii) introduction, (iv) methods, (v) results, (vi) discussion, and (vii) other relevant information. Because the CHEERS checklist evaluates the quality of reporting in economic evaluation studies rather than their quality of its conduct, a qualitative assessment was conducted to examine the completeness of reporting for each item in the included studies. No formal assessment of certainty in the body-of-evidence was undertaken.

## 3. Results

### 3.1. Literature Search Results

**Figure 1:**
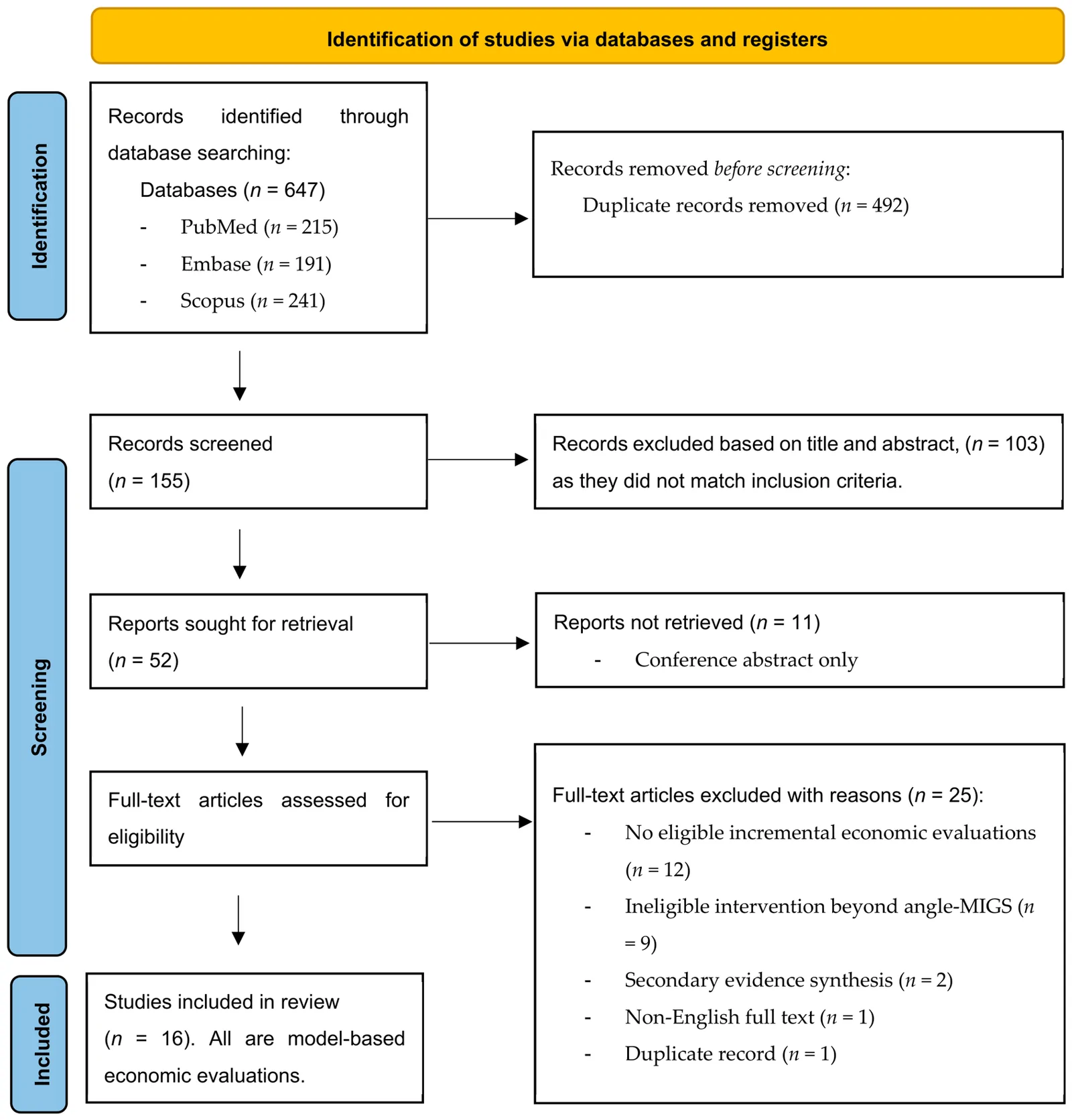
PRISMA Flow Diagram Depicting the Literature Search and Study Selection Process. Note: PRISMA = Preferred Reporting Items for Systematic Reviews and Meta-Analyses. The flow diagram illustrates the systematic process of identification, screening, eligibility appraisal, and final inclusion of economic evaluations for the synthesis.

The combined searches of all databases yielded 647 studies, of which 492 duplicates were removed prior to screening, leaving a total of 155 studies. After preliminary screening of titles and abstracts, another 103 studies were excluded as they did not meet the inclusion criteria. A total of 41 studies then remained for full-text review and were evaluated for eligibility. Ultimately, 16 studies were judged eligible for inclusion in the review, published between 2018 to 2025 (Figure 1, Table 1).

### 3.2. Study Characteristics

**Figure 2:**
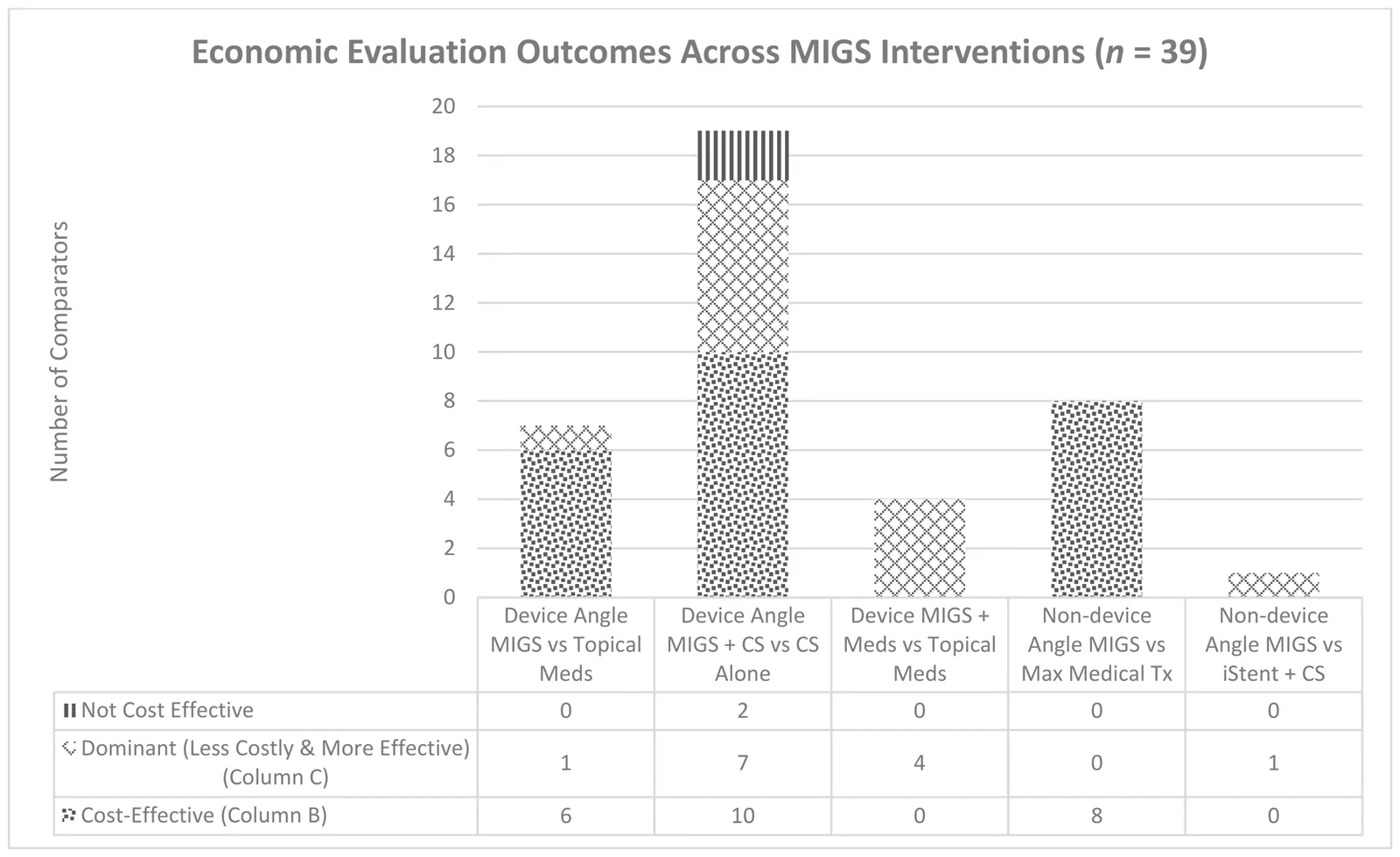
Structured synthesis of the direction of evidence of economic evaluation studies comparing intervention with comparison groups.

These studies included patients from the US (*n* = 3), Canada (*n* = 3), Brazil (*n* = 2), Italy (*n* = 1), Spain (*n* = 1), Germany (*n* = 1), France (*n* = 1), Australia (*n* = 1), Japan (*n* = 1), Colombia (*n* = 1) and China (*n* = 1). Of the 39 economic evaluations performed within these 16 studies, outcomes were analyzed from the payer (*n* = 11), healthcare system (*n* = 20), and societal (*n* = 8) perspectives, the latter incorporating indirect and non-medical costs such as productivity loss, informal caregiver burden, and patient transportation (Table 2). The majority of the economic evaluations assessed the cost-effectiveness of angle-based MIGS with or without CS as compared to CS alone, medical, or laser therapy (Figure 2). These evaluations comprised 19 comparisons of device-based angle MIGS and CS against CS alone, 8 comparisons of non-device-based angle MIGS against maximal medical therapy, 7 comparisons of device-based angle MIGS against topical medication, 4 comparisons of device-based angle MIGS and topical medication against topical medication only, and 1 comparison of non-device based angle MIGS against device-based angle MIGS (Table 3).

### 3.3. Methodology Analysis

Across the evaluated comparisons, all studies utilized decision-analytic modelling to estimate long-term cost-effectiveness. All the evaluations relied on health-state transition Markov models. Time horizons ranged from 1 year to lifetime (i.e., across the population’s estimated natural lifespan), and the discount rates ranged from 1.50% to 5.00% (Table 1). All studies reported QALY as their primary outcome measure, with a majority also estimating the resulting ICER and ICUR.

### 3.4. Quality Assessment

Methodological quality was evaluated using the 10-item Drummond checklist, with overall scores ranging from 8.0 to 9.5 out of a maximum score of 10, reflecting generally average-to-good methodological rigor across the included studies. All studies reported clear decision problem formulations, applied standard annual discount rates (3.0–3.5%), and evaluated uncertainty through sensitivity analyses (both deterministic and probabilistic). High-scoring studies relied randomized controlled trial data or meta-analyses to model IOP lowering and medication reduction. However, long-term models (>10 years or lifetime horizons) required assumptions regarding annual rate of efficacy waning (up to 10% loss of treatment effect) (Table 4 and Table 5).

The item-level CHEERS 2022 assessment is presented in Table 6. Reporting was generally complete for the study objectives, settings, comparators, economic perspective, time horizon, and main economic results. No included study reported whether a health economic analysis plan had been developed or where it could be accessed. Reporting of heterogeneity, distributional effects, and stakeholder engagement was inconsistent.

### 3.5. Modelling Framework and Disease States

All cost-effectiveness analyses reconstructed visual field decline and progression of severity utilizing Markov or semi-Markov cohort simulation models. Patients progressed along discrete, irreversible POAG health states categorized according to visual field mean deviation (MD) loss on the Hodapp-Parrish-Anderson (HPA) scale, comprising mild POAG (0 to −6 dB MD loss), moderate POAG (−6.01 to −12 dB MD loss), advanced POAG (−12.01 to −20 dB MD loss), severe POAG or blindness (<−20 dB MD loss), and death as the final state. Transition probabilities between these progressive glaucoma states were calculated based on the baseline natural visual field progression rate reported in the Early Manifest Glaucoma Trial (EMGT) [52], representing a decline of −0.05 dB per month for untreated patients. Transition probability values were adjusted dynamically or year-by-year based on treatment-specific IOP reductions, applying the EMGT-derived principle that each 1 mmHg decrease in IOP slows the rate of visual field deviation progression by 9.5%. Models incorporated clinical success and failure probabilities, weaning or erosion of treatment efficacy (declining by 5% to 10% annually after the trial window), and subsequent treatment lines (such as SLT or trabeculectomy) upon disease progression.

### 3.6. Primary Outcomes

Overall, angle-MIGS interventions demonstrated favourable economic profiles across all 39 evaluated comparisons, being categorized as either cost-effective (*n* = 24, 61.5%), economically dominant (*n* = 13, 33.3%) or not cost-effective (*n* = 2, 5.1%). Economic dominance indicates greater health gains at lower overall costs [53].

#### 3.6.1. Device-Based Angle MIGS with CS Versus CS Alone

There was a total of 19 economic evaluations spanning 11 studies across 8 countries of combined device-based angle MIGS and CS versus CS alone, analyzed from either a public payer (*n* =16 evaluations across 9 studies) or societal (*n* = 3 evaluations across 2 studies) perspective. For combined procedures (e.g., Hydrus + CS, iStent/iStent inject + CS), 17 comparisons favoured the combined MIGS procedure (Cost-effective: *n* = 10 Dominant: *n* = 7); 2 comparisons involving patients with advanced POG were found not cost-effective at the stated WTP. Of note, all 8 of these countries (Canada, Australia, Japan, USA, Italy, France, Spain, Germany) were considered high-income countries by the World Bank [54]. These findings suggest that adding device-based angle MIGS to CS may represent a cost-effective option in selected populations and healthcare settings, rather than across all disease stages.

#### 3.6.2. Device-Based Angle MIGS Versus Medical Therapy

Economic evaluations comparing standalone device-angle MIGS (iStent standalone) against topical medication consistently favoured the MIGS intervention, with seven evaluated comparisons categorizing the device as either cost-effective (*n* = 6) or economically dominant (*n* = 1). These comparisons involved 3 high- and upper-middle-income countries (Australia, Brazil and Canada) across 4 studies. In Brazil, Paletta et al. (2022, Brazil) [39] evaluated standalone iStent inject implantation over a lifetime horizon from the public healthcare payer perspective of the Brazilian Unified Health System (SUS), capturing direct medical costs. Under the base-case scenario reflecting the SUS Clinical Protocol and Therapeutic Guidelines (PCDT) perspective, the intervention yielded an incremental gain of 0.47 QALYs and an ICER of R$7996.66/QALY compared to topical medications over a lifetime horizon (+0.47 QALYs gained). When modelled using real-world treatment patterns from the Datasus database, the ICER improved to R$4485.68/QALY. In Australia, Healey et al. (2022) [38] adopted a public health system payer perspective to compare standalone trabecular bypass device surgery against medical care over a 15-year horizon. They reported an ICER of A$14,644/QALY with an incremental gain of 0.1526 QALYs per patient, a ratio that falls comfortably below the A$50,000/QALY cost-effectiveness thresholds generally accepted by Australia healthcare payers.

#### 3.6.3. Device-Based Angle MIGS with Topical Medications Versus Topical/Laser Therapy

Only 1 study compared the cost-effectiveness of a combined device-based angle MIGS procedure with topical medications against non-surgical options (e.g., topical medications and laser therapy). A total of 4 comparisons were made by Ordóñez et al. (2019, Colombia) [50]. These evaluations, comparing device-based MIGS combined with medication (specifically, iStent implantation combined with Timolol) against topical medication-only group (*n* = 3 comparisons) or laser plus topical medication (*n* = 1 comparison), consistently demonstrated that the strategy incorporating MIGS was dominant (*n* = 4, 100%). Over a lifetime horizon, standalone iStent implantation generated significantly greater utility at the population level, producing US$127,971 more discounted cohort-level QALYs than SLT (with combined Timolol/ Dorzolamide), and between US$378,287 and US$405,982 more QALYs when compared to maximal medication therapy. This surgical intervention achieved greater health benefits at a lower cumulative cost over the population’s lifetime horizon, resulting in negative ICERs ranging from −$104 versus laser trabeculoplasty, and −$59 to −$90 when compared to different multiple-medication regimens, modelled as receiving three active drug classes simultaneously to reflect realistic clinical practice. In contrast, the iStent surgical arm patients were assumed to have reduced their medication burden to a single drop (timolol or latanoprost). These analyses primarily reflected the public payer perspective of the General System of Social Security in Health of Colombia (SGSSS), capturing direct healthcare resource utilization and medication costs from a national Health Maintenance Organization (HMO) invoice database.

#### 3.6.4. Non-Device Based Angle MIGS Versus Device-Based Angle MIGS

There was only 1 study by Longo et al. (2023, US) that compared the OMNI canaloplasty surgical system combined with CS, with the iStent inject and CS, showing that the former was dominant over a lifetime Medicare perspective. Over a patient’s lifetime horizon, the OMNI procedure accumulated US$11,088 in total costs compared to US$11,640 for the iStent inject, resulting in an overall cost savings of $552. Additionally, the OMNI system produced a slight health benefit by yielding 8.95 QALYs versus 8.93 QALYs for the iStent inject. The OMNI surgical system hence achieved a net monetary benefit (NMB) of $1422 at a $50,000 willingness-to-pay threshold assumed in the study.

#### 3.6.5. Non-Device Based Angle MIGS Versus Maximal Medical Therapy

There were a total of 8 economic evaluations that compared non-device based angle MIGS (ABiC and GATT) against maximal medical therapy, defined as maximal tolerated medical treatment (MMT) consisting of a combination of three or more topical ocular hypotensive medications. All comparisons were derived from a single study in China [36], that evaluated both interventions (ABiC and GATT) in urban and a rural population, with a uniform discount rate of 3.5%, from the societal’s perspective over a 5 and 10 year horizon. The WTP thresholds were US$30 501 per QALY for the rural setting and US$41 568 per QALY for urban settings. At the 5-year horizon, both procedures fell below their respective WTP thresholds of US$30,501 per QALY for rural settings and US$41,568 per QALY for urban settings, making them as cost-effective options. Specifically, compared with maximal medical treatment (MMT), the 5-year ICURs for both GATT and ABiC in rural settings were US$26,945 per QALY and US$29,178 per QALY, respectively. In urban settings, the procedures demonstrated even more favorable economic profiles, with ICURs of US$22,400 per QALY for GATT and US$24,619 per QALY for ABiC. Over a 10-year horizon, the cost-effectiveness of both surgical options improved substantially. In the rural cohort, the ICUR fell to US$19,619 per QALY for GATT and US$18,003 per QALY for ABiC; notably, at 10 years, ABiC became more cost-effective than both GATT and classic trabeculectomy (US$19,675 per QALY). In the urban cohort, the ICURs reached their highest economic efficiency, falling to US$15,675 per QALY for GATT and US$14,203 per QALY for ABiC.

## 4. Discussion

This systematic review synthesized 39 cost-effectiveness comparisons of angle-based MIGS, performed alone or combined with CS, and showed these procedures to be consistently cost-effective across both short-term and lifetime horizons (up to 51.3% of modelled scenarios). Evaluated primarily from payer/healthcare-system, and, less commonly, across payer and societal perspectives, the included models generally reported favorable economic outcomes, although the direction and magnitude of findings varied by comparison and disease stage. These findings support angle-based MIGS as an economically viable alternative or adjunct to current treatment modalities for mild-to-moderate POAG. However, the majority of current research conducted relied on simulated data from randomized controlled trials rather than real world data. Furthermore, there is a notable lack of cost-effectiveness analyses for angle-based MIGS in middle-low-income settings, as well as limited comparisons with more recent glaucoma therapies like laser trabeculoplasty. In this regard, further research is needed to address these knowledge gaps.

While the primary focus of this review is on economic outcomes, it is also important to interpret the findings in relation to the clinical evidence and assumptions underpinning each model. For example, Nieland et al. (2021) incorporated IOP reduction and postoperative medication use from the two-year iStent inject pivotal trial, together with estimates of glaucoma-progression and health-related utilities, to project lifetime costs and QALYs [45]. In contrast, Ahmed et al. (2025) used annualized visual-field progression rates derived from a post-hoc analysis of five-year HORIZON trial data to inform a 15-year economic model of Hydrus implantation combined with cataract surgery [35]. These examples demonstrate how clinical outcomes informed economic evaluations, although benefits extending beyond the available clinical follow-up remained dependent on modelling assumptions. As such, the economic findings should be interpreted within the populations, procedures and healthcare settings evaluated, rather than as evidence of equivalent long-term clinical benefits across all MIGS procedures or patient groups. Their application to treatment decisions should therefore be considered alongside procedure-specific evidence on clinical-effectiveness, safety and patient suitability.

The primary driver of long-term cost-effectiveness across the reviewed models was the reduction in chronic ocular hypotensive medication burden and delayed progression to advanced visual field loss or blindness. Initial surgical and device acquisition costs (e.g., iStent inject or Hydrus Microstent) were typically offset over 3.5 to 5 years. In European health system models, such as France and Italy, the incremental cost per QALY gained remained well below regional WTP thresholds, ranging from €1154/QALY to €13,037/QALY [44,45]. Evidence from German disease-transition models indicated that intervening during moderate POAG maximized health utility gains and cost-utility relative to delaying surgery until advanced disease stages (e.g., €10,955/QALY in moderate POAG vs. €60,269.14/QALY in advanced POAG for two microbypass stents) [47]. Together, these analyses showed that device-based MIGS and combined CS-MIGS were cost-effective and in a substantial proportion of comparisons, cost-dominant, relative to medical therapy and CS alone, providing the quantitative basis for considering these procedures as viable alternatives mild-moderate POAG intervention modalities.

Notably, out of 19 modelled comparisons from 10 studies across eight countries evaluated comparing device-based MIGS combined with CS versus CS alone, 17 comparisons (89.5%) showed that device-based MIGS in combination with CS was either dominant or cost-effective when compared to CS alone. Only two comparisons, both from Bartelt-Hofer et al., exceeded the WTP threshold and did not favor MIGS as a cost-effective intervention in advanced POAG. Because advanced POAG is not a primary clinical indication [55] for standard trabecular micro-bypass, the non-cost-effective outcomes corroborate current clinical consensus that angle-based MIGS may not suited for end-stage glaucomatous disease [56]. Furthermore, these two non-cost-effective comparisons specifically evaluated only the initial generation (e.g., one- and two-stent models) of the iStent. This distinction is important because findings from evaluations of one or two trabecular micro-bypass stents should not be extrapolated to newer systems such as iStent infinite, which incorporates 3 separate trabecular bypass stents. Although clinical studies have reported IOP and medication reductions with this newer system, no eligible device-specific economic evaluation was identified in this review, leaving its cost-effectiveness unestablished [57]. Furthermore, these studies were conducted across high-income countries. The dominance and cost-effectiveness of a combined MIGS-CS procedure could be explained by the fact that the concomitant use of device-based MIGS with CS may help offset operating room and facility costs that would otherwise make standalone MIGS financially burdensome [58]. Furthermore, greater cost-effectiveness might also have been realized through increased clinical efficacy of the combined procedure, thereby reducing dependence on topical drops and decreasing the frequency of follow-up clinic visits compared to CS alone [59]. As such, the scarcity of evaluations from low- and middle-income countries is a genuine generalizability limitation of the device-based MIGS literature that warrants further exploration.

Because non-device angle-based MIGS procedures such as GATT and ab interno canaloplasty eliminate device-related expenditures, these procedures are expected to be more cost-effective, especially in resource-limited healthcare settings where medication non-adherence and loss to follow-up are prevalent issues [60,61]. However, in the included Chinese economic evaluation, incremental QALY gains were similar between rural and urban settings, despite differences in estimated costs and ICURs [36]. The similarity in values could have been explained by the assignation of standardized utility weights to specific glaucoma stages within the Markov model utilized to estimate cost-effectiveness in this study. As such, further evaluation incorporating local resource use, access to care and long-term clinical outcomes are needed to assess potential rural-urban disparities in cost-effectiveness across the various healthcare settings.

Extensive research has shown that angle-based MIGS procedures are effective in lowering IOP for the management of glaucoma [62,63]. Our research supplements these clinical efficacy data, and suggests that angle-based MIGS procedures are cost-effective or economically dominant in mild-moderate POAG populations and in the healthcare settings studied, relative to medical therapy or CS alone. The only two comparisons that were classified as not cost-effective involved advanced POAG, highlighting the importance of disease stage when interpreting economic value. While further real-world and low-income setting data are warranted, these findings advocate for the consideration of selected angle-based MIGS procedures as a viable primary treatment options for mild-to-moderate POAG in high-resource settings. This aligns seamlessly with the broader clinical movement toward “interventional glaucoma” [64,65,66], a treatment paradigm that prioritizes interventions like MIGS, lasers, and sustained drug delivery systems early in the disease over first-line topical therapy. However, we must acknowledge the paucity of data comparing MIGS against newer treatment modalities, such as triple-combination topical drops, selective laser trabeculoplasty (SLT), and micropulse transscleral cyclophotocoagulation (MPTCP). The predominantly model-based evidence and concentration of device-based evaluations in high-income settings warrant caution when extending these conclusions to other healthcare systems. Decisions regarding reimbursement or broader adoption should also be consider local costs, affordability, service capacity and uncertainty surrounding long-term clinical benefits. Further health economic studies may be warranted to determine if MIGS is still economically dominant against these newer interventions.

Strengths of this review include a comprehensive synthesis of published literature in a standardized and systematic manner that encompasses diverse geographic settings and incorporates both payer and societal perspectives. However, several limitations must be acknowledged. The literature search was restricted to MEDLINE/ PubMed, Embase and Scopus, which were selected to capture literature relevant to glaucoma interventions and economic evaluations. As a result, our search was not exhaustive across all literature sources, and economic evaluations indexed exclusively in other databases or available through health technology assessment sources may have been missed. Furthermore, the use of cataract-related search terms may have reduced sensitivity for standalone MIGS evaluations, potentially limiting the completeness of the evidence identified for these procedures. Besides, the data was extracted by one reviewer (TST) and independently verified by a second reviewer (SST), rather than extracted independently in duplicate. Although verification provided and additional check on accuracy, this approach may have left residual extraction errors, omissions or differences in interpretation. Currently, all 19 identified device-based MIGS comparisons evaluated either the Hydrus microstent, or the iStent implant, thus limiting the extent to which the findings can be generalized to other MIGS procedures. Several included economic evaluations also received manufacturer funding or involved authors with relevant financial relationships, raising the possibility of sponsorship bias in the selection of comparators, modeling assumptions or interpretation of results. Although such relationships do not necessarily indicate biased findings, their potential influence should be considered when interpreting the review’s conclusion. Additionally, most of the included studies relied heavily on estimated transition probabilities from simulated clinical trial datasets, rather than prospective real-world clinical data, further limiting data generalizability. The applicability of these findings depends on both the procedure evaluated and the characteristics of the clinical population and modelled cohort, including glaucoma severity, baseline IOP, medication burden, previous treatment and disease-progression rate. Regional cost structures, and varying assumptions regarding medication adherence may have heavily influenced the magnitude of cost savings in decision making. Given these clinical and methodological differences across the included studies, the outcomes of this review should be interpreted within specific patient populations and healthcare settings, rather than evidence that all MIGS procedures are uniformly cost-effectiveness compared with other glaucoma interventions. Despite these limitations however, the robust and uniform direction of the synthesized evidence strongly supports the conclusion that MIGS is a cost-effective glaucoma surgical procedure in the context of the clinical population and settings examined.

## 5. Conclusions and Future Directions

In conclusion, angle-based MIGS is a cost-effective intervention that provides value in the management of mild-moderate glaucoma, particularly in high-income clinical settings. The initial upfront procedural costs of MIGS are likely to be mitigated over time by the reduction in medication dependence and a slower disease progression resulting in greater cost-effectiveness compared to currently recommended glaucoma intervention modalities. These robust economic findings of MIGS further supports its proactive use in interventional glaucoma as a financially sound alternative to lifelong topical medications and standalone CS. To optimize healthcare resource allocation, future research must prioritize long-term head-to-head economic evaluations between different MIGS classes and robust real-world implementation studies, particularly in lower-resource settings.

## Figures and Tables

**Table 1:** Basic Characteristics of Included Studies.

Authors	Year	Mean Age (SD)	Glaucoma Severity	Country/ Perspective(s)	Main Comparison (Intervention vs. Comparator)	Time Horizon(s)	Main Research Variable(s)	Cost Year, Currency, WTP Threshold	Discount Rate	Sensitivity Analysis
Ahmed et al. [35]	2025	71 (NR)	Mild-to-moderate POAG	Canada Payer, Societal	Hydrus + CS vs. CS Alone	15 yrs	QALY, ICUR	2023 CAD, C$50,000	1.50%	PSA, DSA
Li et al. [36]	2023	40 (NR)	Mild-to-moderate POAG	China (Urban, Rural), Societal	GATT/ABIC vs. MMT	5, 10 yrs	QALY, ICUR	2021 USD Rural setting: US$30,501 Urban setting: US$41,568	3.50%	PSA, DSA
Longo et al. [37]	2023	65 (NR)	Mild-to-moderate POAG	US, Payer (Medicare)	OMNI Canaloplasty vs. iStent inject^®^ + CS	Lifetime	QALY, NMB	2023 USD $50,000	3.00%	PSA, DSA
Healey et al. [38]	2022	73 (8)	Mild-to-moderate OAG	Australia, Payer	iStents vs. medical treatment and iStents + CS vs. CS Alone	15 years	QALY, ICER	2017 AUD A$50,000	5.00%	DSA
Paletta Guedes et al. [39]	2022	63 (NR)	Mild-to-moderate POAG	Brazil, Payer	iStents vs. medical treatment	Lifetime	QALY, ICER, ICUR	2020 BRL R$94,761 (eq. 3× GDP)	5.00%	PSA, DSA
Igarashi et al. [40]	2022	64.5 (NR)	Mild-to-moderate POAG	Japan, Payer, Societal	iStents + CS vs. CS Alone	Lifetime	QALY, ICER, ICUR	2021 JPY ¥5,000,000	2.00%	PSA, DSA
Sood et al. [41]	2022	65 (NR)	Mild-to-moderate POAG	US, Societal	Hydrus + CS vs. CS Alone	35 years	QALY, ICER	2021 USD $50,000	3.00%	PSA, DSA
Atik et al. [42]	2022	65 (NR)	Mild-to-moderate POAG	US, Payer, Societal	Hydrus + CS vs. Medical treatment post-CS	2, 10 yrs	QALY, ICUR	2021 USD $50,000	3.00%	PSA, DSA
Paletta Guedes et al. [43]	2021	63 (NR)	Mild-to-moderate POAG	Brazil, Payer	iStents standalone vs. Medical Treatment	Lifetime	QALY, ICER	2021 BRL 1–3× GDP per capita	5.00%	PSA, DSA
Fea et al. [44]	2021	64.7 (12.1)	Mild-to-moderate POAG	Italy, Payer	iStents + CS vs. CS Alone	Lifetime	QALY, ICER, ICUR	2021 EUR €25k–€40k	3.50%	PSA, DSA
Nieland et al. [45]	2021	65 (NR)	Mild-to-moderate OAG	France, Societal	iStents + CS vs. CS Alone	Lifetime	QALY, ICER	2019 EUR €30,000	4.00%	PSA, DSA
Teus et al. [46]	2021	63.8 (NR)	Mild-to-moderate POAG	Spain, Societal	iStent + CS vs. CS Alone	Lifetime	QALY, ICER	2020 EUR €21k–€30k	3.00%	PSA, DSA
Bartelt-Hofer et al. [47]	2020	63 (NR)	Moderate -Advanced POAG	Germany, Payer	iStent (1) + CS/iStents (2) + CS vs. CS Alone	Lifetime	QALY, ICER	2019 EUR €43,433.87	3.00%	PSA, DSA
Patel et al. [48]	2019	65 (NR)	Mild-to-moderate OAG	Canada, Payer	iStents standalone vs. Medication Alone	15 yrs, NA	QALY, ICER, ICUR	2017 CAD $50,000	1.50%	PSA, DSA
Ahmed et al. [49]	2019	65 (NR)	Mild-to-moderate OAG	Canada, Payer, Societal	iStents + CS vs. CS Alone	15 yrs, NA	QALY, ICER, ICUR	2017 CAD C$50,000	1.50%	PSA, DSA
Ordóñez et al. [50]	2018	40 (NR)	Mild-to-moderate POAG	Colombia, Societal	iStents + Timolol vs. Triple Therapies and/or SLT	Lifetime	QALY	2017 USD 1–3× GDP per capita	5.00%	PSA, DSA

WTP: willingness to pay; CS: cataract surgery; QALY: quality-adjusted life years; ICUR: incremental cost-utility analysis; PSA: probabilistic sensitivity analysis; DSA: deterministic sensitivity analysis; GATT: gonioscopy-assisted transluminal trabeculotomy; ABIC: ab interno canaloplasty; MMT: maximal medical therapy; GDP: gross domestic product; NMB: net monetary benefit; ICER: incremental cost-effectiveness ratio; ICUR: incremental cost-utility ratio; US: United States.

**Table 2:** Key Indices and Outcome Statistics for Included Economic Evaluations.

Author (Years)	Intervention Evaluated	Clinical Inputs	Costs	QALYs	ICER/ICUR	Key Remarks/Fundings Summary
Ahmed et al. (2025)	Hydrus Microstent	Annual VF RoP (0.26 dB/yr vs. 0.49 dB/yr) mapped to drops	Base case: Hydrus + CS: C$26,770 CS: C$27,145 Societal perspective: Hydrus + CS: C$26,828 CS: 27,411	Hydrus + CS: 9.351 CS: 9.040 Incremental QALY: 0.311	Dominant	Hydrus + CS dominated CS alone in base case and in most scenario analyses, with an 85.3% probability of being cost-effective at Can$50,000/QALY
Li et al. (2023)	GATT ABiC	QALYs gained; Markov state transitions based on POAG progression	Rural setting: Base case (5 years): GATT: US$9848 ABiC: US$10,471 MMT: US$3570 Base case (10 years): GATT: US$12,613 ABiC: US$13,598 MMT: US$6458 Urban setting: Base case (5 years): GATT: US$8317 ABiC: US$8920 MMT: US$3117 Base case (10 years): GATT: US$10,649 ABiC: US$11,366 MMT: US$5761	Rural setting: 5 years: GATT: 3.37 ABiC: 3.38 MMT: 3.14 Incremental QALYs: GATT−MMT: 0.23 ABiC−MMT: 0.24 10 years: GATT: 5.97 ABiC: 6.05 MMT: 5.65 Incremental QALYs: GATT−MMT: 0.31 ABiC−MMT: 0.40 Urban setting: 5 years: GATT: 3.37 ABiC: 3.38 MMT: 3.14 Incremental QALYs: GATT−MMT: 0.23 ABiC−MMT: 0.24 10 years: GATT: 5.97 ABiC: 6.05 MMT: 5.66 Incremental QALYs: GATT−MMT: 0.31 ABiC−MMT: 0.39	ICUR Rural setting: 5 years: GATT: US$26,945 ABiC: US$29,178 10 years: GATT: US$19,619 ABiC: US$18,003 Urban setting: 5 years: GATT: US$22,400 ABiC: US$24,619 10 years: GATT: US$15,675 ABiC: US$14,203	Evaluated GATT & ABIC produced lower ICURs than trabeculectomy in rural areas. Both interventions are more cost-effective than MMT across urban and rural sub-settings over 5 and 10 yrs.
Longo et al. (2023)	OMNI vs. iStent inject	6-month DIOP reduction (37.8% OMNI vs. 37.1% iStent inject)	Lifetime horizon OMNI^®^: $11,088 iStent inject^®^: $11,640	OMNI^®^: 8.95 iStent inject^®^: 8.93 Incremental QALYs: +0.02	Dominant	OMNI dominates iStent inject (Lower overall cost, higher QALY gains). OMNI was cost-saving relative to iStent inject^®^ + CS
Healey et al. (2022)	TBD with and without CS	IOP reduction (10% progress reduction per 1 mmHg drop)	TBD + CS: A$7946 CS: A$7770 TBD: A$6594 Usual care: A$4360	TBD + CS: 6.5095 CS: 6.4369 TBD:7.339 Usual care: 7.1864 Incremental QALYs: TBD + CS vs. CS: +0.0726 TBD vs. usual care: +0.1526	ICER: TBD + CS vs. TBD: A$2430 TBD vs. usual care: A$14,644	TBD is a cost-effective intervention for POAG in the Australian setting.
Paletta Guedes et al. (2022)	iStent inject vs. medications	QALYs gained; local clinical practice guidelines and real-world compliance	Base case (PCDT): iStent inject: BRL 12,327.43 Medications: BRL 8564.02 Real world (Datasus): iStent inject: BRL 13,606.28 Medications: BRL 11,520.39	Base case (PCDT): iStent inject: 10.09 Medications: 9.62 Real world (Datasus): iStent inject: 10.09 Medications: 9.63	Base case (PCDT): iStent inject vs. medications: BRL 7996.66 Real world (Datasus): iStent inject vs. medications: BRL 4485.68	Compared real-world settings vs. CPTG Guidelines; focused on progression. iStent inject is considered cost-effective compared to topical medication.
Igarashi et al. (2022)	iStent inject	RCT-derived IOP reduction & drop count, dB transition model	Base case: iStent inject + CS: ¥1,025,785 CS: ¥933,759 Scenario analaysis iStent inject + CS: ¥6,846,877 CS: ¥7,055,679	Base case: iStent inject + CS: 12.8 CS: 12.74 Incremental QALYs: 0.06 Scenario analysis iStent inject + CS: 12.833 CS: 12.767 Incremental QALYs: +0.066	Base case: ICER: ¥12,845,154/blind eye avoided ICUR: ¥1,430,647/QALY Scenario analysis: ICER and ICUR: Dominant WTP threshold: ¥5,000,000 per QALY gained	First Japanese study evaluating iStent; dominant in societal perspective. Public Payer Perspective: Incremental cost of ¥92,026 and QALY gain of 0.06 yielded an ICUR of ¥1,430,647/QALY gained. Societal Perspective: iStent inject^®^ with cataract surgery was dominant (produced a cost saving of −¥208,803 and a QALY gain of 0.066).
Sood et al. (2022)	Hydrus Microstent vs. iStent (combined with CS) vs. CS alone vs. medication	QALYs gained; reduction in IOP, drop burden, and progression to severe visual field loss	Base case: Hydrus + CS: $33,813.10 iStent inject + CS: $35,328.02 CS alone: $39,724.82 Meds: $57,931.22 Scenario analysis: Hydrus + CS: $48,026.13 iStent inject + CS: $49,599.86 CS alone: $54,409.25 Meds: $57,931.22	Base case: Hydrus + CS: 9.54 iStent inject + CS: 9.51 CS alone: 9.42 Scenario analysis: Hydrus + CS: 11.41 iStent inject + CS: 11.35 CS alone: 11.14	Base case: ICER 5-year horizon: iStent inject + CS: $39,670.93 Scenario analysis: Hydrus + CS: $3175.09 iStent inject + CS: $6458.24	Both stent arms dominated cataract surgery alone and medication management within 5 years. Hydrus slightly dominated iStent inject^®^.
Atik et al. (2022)	Hydrus Microstent + CS vs. CS alone	Medicated IOP drop (2.25 mmHg) & medication reduction (0.41 drops)	Base Case: Hydrus + CS: 7002.36 CS: 4884.36	Base Case: Hydrus + CS: 20.95 utilities * CS: 20.90 utilities Incremental utilities: −0.05	Base Case: Hydrus + CS vs. CS Mild glaucoma: ICUR: $38,346.43 Mod glaucoma: ICUR: $42,895.38	Probabilistic sensitivity analysis was cost-effective in 61.4% of iterations for Hydrus + CS. The probability of side-effects with eye drops, utility decrement with side-effects, cost of the Hydrus and real-world efficacy rate had the greatest impact on model outcomes. Clinically, hydrus must be 85.60% as effective as published data to maintain cost-effectiveness at a willingness-to-pay threshold of USD 50,000.
Paletta Guedes et al. (2021)	iStent inject^®^ (Standalone) vs. Topical Medications	QALYs gained, local clinical practice guidelines and real-world compliance	Base case: iStent inject: R$19,856.98 Drug: R$8564.02 Real-world: iStent inject: R$21,135.83 Drug: R$11,520.39	PFLY: Base case: iStent inject: 7.82 Drug: 6.33 Incremental PFLY: +1.5 Real world: iStent inject: 7.82 Drug: 6.33 Incremental PFLY: +1.5	ICER per PFLY gained: Base case: R$7550.97 Real world: R$6429.30	iStent inject^®^, when used after the failure of the first-line therapy, is able to reduce the rate of glaucoma progression at an acceptable cost.
Fea et al. (2021)	iStent inject^®^ + CS vs. CS alone	QALYs gained; IOP reduction and reduced medication dependence	Base case: iStent inject + CS: 8368.51 CS: €7134.71	Base case: iStent inject: 11.114 CS: 11.019 Incremental QALY: +0.095 QALYs	Base case (ICER): iStent inject vs. CS: €13,037.01	Cost-effective: ICER of €13,037.01/QALY gained (well below the €25,000–€40,000 Italian WTP threshold). 96% probability of being cost-effective at €50,000 WTP.
Nieland et al. (2021)	iStent inject^®^ + CS vs. CS alone	QALYs gained; reduced medication usage and delayed disease progression to severe glaucoma/blindness	Base case: iStent inject + CS: 13,949 CS alone: €13,874 (€984 of initial device investment offset by downstream savings)	Base case: iStent inject + CS: 11.05 CS:10.98 Incremental QALY: +0.065 QALYs	Base case: ICER: €1154	Probability of being cost-effective is 86% at a €30,000 WTP threshold. Dominates in non-persistent patients and moderate-disease scenarios.
Teus et al. (2021)	iStent inject^®^ + CS vs. CS alone	QALYs gained; reduction in topical drops and delayed surgical revisions	SNHS: Higher initial cost with partial offset. iStent inject + CS: €13,344.05 CS alone: €12,293.86 Societal: Net cost-saving (€47,446.30 vs. €49,777.60)	SNHS: iStent inject + CS: 12.36 CS: 12.27 Incremental QALY: 0.083	SNHS: ICER €12,676.48/QALY Societal: Dominant (Cost-saving).	High probability of cost-effectiveness within Spanish healthcare framework. SNHS perspective: 90.9% probability cost-effective at €30,000 WTP.
Bartelt-Hofer et al. (2020)	1 TBD vs. 2 TBD vs. IS vs. CS alone	QALYs gained; 1-year IOP reduction from baseline (1 TBD: −3.15 mmHg; 2 TMBSs: −4.85 mmHg; IS: −2.25 mmHg; Phaco alone: −2.05 mmHg)	Moderate POAG: CS alone: 4103.28 1 TBD + CS: 4179.83 2 TBDs + CS: 4240.53 Advanced POAG CS alone: 3917.76 1 TBD + CS: 3947.83 2 TBDs + CS: 3971.11	Moderate POAG: CS alone: 9.72 1 TBD + CS: 9.73 2 TBDs + CS: 9.74 Advanced POAG CS alone: 9.68 1 TBD + CS: 9.68 2 TBDs + CS: 9.68	Moderate POAG: ICER: 1 TBD + CS: €15,673.41 2 TBDs + CS: €10,955.20 Advanced POAG 1 TBD + CS: €86,942.73 2 TBDs + CS: €60,269.14	Superior efficacy for iStent (2) + CS; iStent (1) + CS was less cost-effective than combo. 2 TBDs with CS yielded the highest added value and lowest ICER. Interventions in advanced POAG were far less cost-effective (ICERs > €60,000/QALY)
Patel et al. (2019)	Standalone TBD (2× iStent/1× iStent inject) vs. Medication alone	QALYs gained; reduction in medication dependence	Base case: TBD alone: C$9394.1 Medication: C$12,302.4 Scenario analyses (Lifetime): TBD: C$13,086.4 Medication: C $15,873.8	Base case: TBD: 9.239 Medication: 9.171 Incremental QALY: +0.068 Scenario analyses (Lifetime): TBD: 13.054 Medication: 12.982	Base case: ICER: −C$938,161.3 per blind eye avoided (TBD dominates) ICUR: −C$42,769.1 per QALY (TBD dominates) Scenario analyses (Lifetime): ICER: −C$844,666.7 (TBD alone dominates) ICUR: −C$39,259.2 (TBD alone dominates)	TBD strongly dominated medication alone. Time-to-dominance reached at 44 months (3.7 years).
Ahmed et al. (2019)	iStent inject^®^ + CS vs. CS alone	QALYs gained; reduction in mean number of medications	Base case: TBD + CS: 21,384.10 CS alone: 21,773.10 Societal perspective: TBD + CS: 93,263.80 CS alone: 93,909.40	Base case: TBD + CS: 9.428 CS alone: 9.405 Incremental QALY: 0.023 Societal perspective: TBD + CS: 9.428 CS alone: 9.405 Incremental QALY: 0.023	Base case: ICER: −C$1,302,385 (TBD arm dominates) ICUR: −C$16,691 (TBD arm dominates Societal perspective: TBD arm dominates (95th percentile, 86,638.05)	Dominant/Cost-saving: 73.7% probability of cost-savings; 94.1% probability of ICUR ≤ C$50,000/QALY.
Ordóñez et al. (2018)	Standalone iStent^®^ (+Timolol) vs. SLT vs. Multiple Medication Schemes	QALYs gained, prevention of advanced glaucoma progression	Net cost savings up to $13.25M vs. SLT and $29.15M vs. triple medical therapy (cohort level)	Discounted QALY: iStent + timolol: 2,226,612 *	Dominant	Dominant strategy: iStent^®^ yielded lower overall lifetime costs and higher QALY gains compared to all competitors.

QALY: quality-adjusted life-years; ICER: incremental cost-effectiveness ratio; ICUR: incremental cost-utility ratio; VF: visual field; RoP: rate of progression; CS: cataract surgery; GATT: gonioscopy-assisted transluminal trabeculotomy; ABiC: ab inferno canaloplasty; POAG: primary open angle glaucoma; MMT: maximal medical therapy; DIOP: diurnal intraocular pressure; TBD: trabecular bypass device; CPTG: clinical protocols and therapeutics guidelines; PCDT: Brazilian Clinical Protocol and Therapeutic Guidelines; RCT: randomised controlled trial; WTP: willingness to pay; PFLY: progression-free life-years; SNHS: Spanish National Health System; IS: intracanalicular scaffold; SLT: selective laser trabeculoplasty. * ”utilities”: determined by a standard gamble of utilities on a standard policy scale using a perfect health anchor [51].

**Table 3:** Summary of Structured Synthesis of economic evaluation studies comparing intervention vs. comparator, with direction of evidence.

Serial	No. of Comparisons	CS	Intervention	Comparator	Economic Study Outcomes	Direction of Evidence
1	7	Markov	Device Angle MIGS	Topical Medication	Cost effective *n* = 6, Dominant *n* = 1	Intervention favourable compared to comparator
2	19		Device Angle MIGS + CS	CS Alone	Cost effective *n* = 10, Dominant *n* = 7 Not cost-effective *n* = 2	Intervention favourable compared to comparator
3	4		Device MIGS + medication	Topical Medication	Dominant *n* = 4	Intervention Dominant compared to comparator
4	8		Non device angle MIGS	Maximal Medical Treatment (MMT)	Cost effective *n* = 8 Dominant *n* = 0	Intervention favourable compared to comparator
5	1		Non device angle MIGS	iStent inject^®^ + Cataract Surgery (CS)	Dominant *n* = 1	Intervention Dominant compared to comparator
Overall	39	All Markov with one semi-Markov	MIGs (Device/non device/meds/CS	Medication (topical, MMT), CS, iStent inject^®^	Cost effective *n* = 24, Dominant *n* = 13 Not cost-effective *n* = 2	Intervention favourable compared to comparator

**Table 4:** Quality Evaluation by Drummond Checklist.

Drummond Criteria	Ahmed (2025)	Li (2023)	Longo (2023)	Healey (2022)	Paletta (2022)	Igarashi (2022)	Sood (2022)	Atik (2022)	Paletta (2021)	Fea (2021)	Nieland (2021)	Teus (2021)	Bartelt-Hofer (2020)	Patel (2019)	Ahmed (2019)	Ordóñez (2018)
Well-defined question?	1	1	1	1	1	1	1	1	1	1	1	1	1	1	1	1
Adequate description of comparators?	0.5	0.5	0.5	0.5	0.5	0.5	0.5	0.5	0.5	0.5	0.5	0.5	0.5	0.5	0.5	0.5
Evidence of effectiveness?	1	0.5	0.5	0.5	0	0	1	1	0.5	0.5	0.5	0.5	1	1	0.5	0.5
Relevant costs/consequences?	1	1	1	0.5	1	0.5	1	1	0.5	0.5	1	1	0.5	1	1	1
Costs/consequences accurately measured?	1	1	1	1	1	1	1	1	1	1	1	1	1	1	1	1
Were the valuation costs/consequences credible?	1	1	1	1	1	1	1	1	1	1	1	1	1	1	1	0.5
Was discounting used as appropriate?	1	1	1	1	1	1	1	1	1	1	1	1	1	1	1	1
Were incremental analyses appropriately reported?	1	1	1	1	1	1	1	1	1	1	1	1	1	1	1	1
Were sensitivity analyses reported?	1	1	1	1	1	1	1	1	1	1	1	1	1	1	1	1
Was the discussion adequate?	1	1	1	1	1	1	1	1	1	1	1	1	1	1	1	0.5
Score out of 10	9.5	9	9	8.5	8.5	8.0	9.5	9.5	8.5	8.5	9	9	9	9.5	9	8

Note: Scores are evaluated on a 10-point scale based on the Drummond 10-item checklist for economic evaluations. Items are scored as 1 (Fully met), 0.5 (Partially met), or 0 (Not met/Not reported). Total scores range from 0 to 10, with higher scores reflecting superior methodological quality and reporting rigor.

**Table 5:** Quality Appraisal of Included Studies Based on the Drummond Criteria.

Study	Drummond Score	Key Strengths & Methodological Limitations
Ahmed (2025)	9.5	High overall quality; clear reporting of effectiveness, credible valuation of costs, and robust sensitivity analyses.
Li (2023)	9.0	Clear clinical question and robust modeling framework; minor limitation in full comparator cost itemization.
Longo (2023)	9.0	Well-structured incremental economic evaluation; limited detail on specific long-term comparator resource use.
Healey (2022)	8.5	Sound decision-analytic model; moderate limitations in comparator descriptions and indirect effectiveness inputs.
Paletta (2022)	8.5	Appropriate time horizons and discounting; relies heavily on indirect effectiveness parameters.
Igarashi (2022)	8.0	Satisfactory core economic reporting; lower score due to limited cost valuation transparency and indirect efficacy sources.
Sood (2022)	9.5	Robust cost measurement and reporting; minor gaps in detailed comparator long-term follow-up protocols.
Atik (2022)	9.5	Comprehensive 2-year Markov model; detailed Medicare cost breakdowns and thorough probabilistic evaluations.
Paletta (2021)	8.5	Well-executed incremental cost-utility analysis; partial itemization of downstream comparator resource costs.
Fea (2021)	8.5	Robust Markov state-transition structure; relied on clinical trial assumptions for long-term efficacy waning.
Nieland (2021)	9.0	Comprehensive French societal perspective; well-validated HCRU inputs and extensive scenario analyses.
Teus (2021)	9.0	The research question, cost and consequence assessment, discounting, incremental analysis, sensitivity analysis and discussion criteria were fully met. Comparator description and evidence of effectiveness were partially met.
Bartelt-Hofer (2020)	9.0	Evidence of effectiveness, measurement and valuation of costs and consequences, discounting, incremental analysis, sensitivity analysis and discussion criteria were fully met. Comparator description and identification of relevant costs and consequences were partially met.
Patel (2019)	9.5	All criteria were fully met except the description of comparators, which was partially met. Strengths included evidence of effectiveness, cost and consequence assessment, discounting, incremental analysis and sensitivity analysis.
Ahmed (2019)	9.0	The research question, cost and consequence assessment, discounting, incremental analysis, sensitivity analysis and discussion criteria were fully met. Comparator description and evidence of effectiveness were partially met.
Ordóñez (2018)	8.0	The research question, identification and measurement of costs and consequences, discounting, incremental analysis and sensitivity analysis criteria were fully met. Comparator description, evidence of effectiveness, credibility of cost/consequence valuation and adequacy of discussion were partially met.

**Table 6:** Completeness Evaluation by the CHEERS Checklist.

CHEERS Item	Ahmed 2025	Li 2023	Longo 2023	Healey 2022	Paletta Guedes 2022	Igarashi 2022	Sood 2022	Atik 2022	Paletta Guedes 2021	Fea 2021	Nieland 2021	Teus 2021	Bartelt-Hofer 2020	Patel 2019	Ahmed 2019/2020	Ordóñez 2018
Title	**1**	**1**	**1**	**1**	**1**	**1**	**1**	**1**	**1**	**1**	**1**	**1**	**1**	**1**	**1**	**1**
Abstract																
Abstract	**1**	**1**	**1**	**1**	**1**	**1**	**1**	**1**	**1**	**1**	**1**	**1**	**1**	**1**	**1**	**1**
Introduction																
3. Background and objectives	**1**	**1**	**1**	**1**	**1**	**1**	**1**	**1**	**1**	**1**	**1**	**1**	**1**	**1**	**1**	**1**
Methods																
4. Health economic analysis plan	**0**	**0**	**0**	**0**	**0**	**0**	**0**	**0**	**0**	**0**	**0**	**0**	**0**	**0**	**0**	**0**
5. Study population	1	1	1	1	1	1	1	1	1	1	1	1	1	1	1	1
6. Setting and location	1	1	1	1	1	1	1	1	1	1	1	1	1	1	1	1
7. Comparators	1	1	1	1	1	1	1	1	1	1	1	1	1	1	1	1
8. Perspective	1	1	1	1	1	1	1	1	1	1	1	1	1	1	1	1
9. Time horizon	1	1	1	1	1	1	1	1	1	1	1	1	1	1	1	1
10. Discount rate	1	1	1	1	1	1	1	1	1	1	1	1	1	1	1	1
11. Selection of outcomes	1	1	1	1	1	1	1	1	1	1	1	1	1	1	1	1
12. Measurement of outcomes	1	1	1	1	1	1	1	1	1	1	1	1	1	1	1	1
13. Valuation of outcomes	1	1	1	1	1	1	1	1	1	1	1	1	1	1	1	1
14. Measurement and valuation of resources and costs	1	1	1	1	1	1	1	1	1	1	1	1	1	1	1	1
15. Currency, price date and conversion	1	1	1	1	1	1	1	1	1	1	1	1	1	1	1	0
16. Rationale and description of model	1	1	1	1	1	1	1	1	1	1	1	1	1	1	1	1
17. Analytics and assumptions	1	1	1	1	1	1	1	1	1	1	1	1	1	1	1	1
18. Characterising heterogeneity	0	1	0	0	1	0	1	1	0	0	1	0	1	0	0	0
19. Characterising distributional effects	0	1	0	0	0	1	0	0	0	0	0	0	0	0	0	0
20. Characterising uncertainty	1	1	1	1	1	1	1	1	1	1	1	1	1	1	1	1
21. Approach to engagement	0	1	1	0	0	1	1	0	0	1	1	1	0	0	1	1
Results																
22. Study parameters	**1**	**1**	**1**	**1**	**1**	**1**	**1**	**1**	**1**	**1**	**1**	**1**	**1**	**1**	**1**	**1**
23. Summary of main results	1	1	1	1	1	1	1	1	1	1	1	1	1	1	1	1
24. Effect of uncertainty	1	1	1	1	1	1	1	1	1	1	1	1	1	1	1	1
25. Effect of engagement	NA	NA	1	NA	NA	1	1	NA	NA	1	1	1	NA	0	1	1
Discussion																
26. Study findings, limitations and generalisability	**1**	**1**	**1**	**1**	**1**	**1**	**1**	**1**	**1**	**1**	**1**	**1**	**1**	**1**	**1**	**1**
Other information																
27. Source of funding	**1**	**1**	**1**	**1**	**1**	**1**	**1**	**1**	**1**	**1**	**1**	**1**	**1**	**1**	**1**	**1**
28. Conflicts of interest	1	1	1	1	1	1	1	1	1	1	1	1	1	1	1	1

## Data Availability

No new data were created or analyzed in this study. Data sharing is not applicable to this article.

## Supplementary Materials

The following supporting information can be downloaded at: https://www.mdpi.com/article/10.3390/medsci14060664/s1, Chart S1: Keywords search terms; Chart S2: Drummond’s checklist for the critical appraisal of economic evaluations. Table S1: Prisma 2020 checklist.
