# Supplementary material for: Cost-Effectiveness of Angle-Based Minimally Invasive Glaucoma Surgery (MIGS): A Systematic Review of Economic Evaluations
Source: Med Sci (Basel). 2026 Oct 12;14(6):664. doi: 10.3390/medsci14060664 (PMC13707156; doi:10.3390/medsci14060664)
Supplement: Supplementary file 1 [file medsci-14-00664-s001.zip › medsci-4602033-supplementary.pdf]

Supplementary Charts:

Supplementary Chart S1: Keywords search terms

PubMed: PHACO-MIGS + cost-effectiveness

```
(
  "Glaucoma"[Mesh] OR
  "Ocular Hypertension"[Mesh] OR
  glaucoma[tiab] OR
  "primary open angle glaucoma"[tiab] OR POAG[tiab] OR
  "primary angle closure glaucoma"[tiab] OR PACG[tiab] OR
  "angle closure glaucoma"[tiab] OR ACG[tiab] OR
  "normal tension glaucoma"[tiab] OR NTG[tiab] OR
  "low tension glaucoma"[tiab] OR
  "secondary glaucoma"[tiab] OR
  "pseudoexfoliation glaucoma"[tiab] OR PXG[tiab] OR
  "pigmentary glaucoma"[tiab] OR
  "neovascular glaucoma"[tiab] OR
  "uveitic glaucoma"[tiab] OR
  "steroid induced glaucoma"[tiab] OR
  "traumatic glaucoma"[tiab] OR
  "aphakic glaucoma"[tiab] OR
  "pseudophakic glaucoma"[tiab] OR
  "ocular hypertension"[tiab] OR OHT[tiab]
)
AND
(
  "Minimally Invasive Surgical Procedures"[Mesh] OR
  "Trabecular Meshwork/surgery"[Mesh] OR
  "Glaucoma/surgery"[Mesh] OR
  MIGS[tiab] OR
  "minimally invasive glaucoma surgery"[tiab] OR
  "microinvasive glaucoma surgery"[tiab] OR
  "minimally invasive glaucoma surgeries"[tiab] OR
  iStent[tiab] OR "iStent inject"[tiab] OR "iStent infinite"[tiab] OR
  Hydrus[tiab] OR "Hydrus microstent"[tiab] OR
  Kahook[tiab] OR "Kahook dual blade"[tiab] OR KDB[tiab] OR
  Trabectome[tiab] OR "ab interno trabeculectomy"[tiab] OR
  OMNI[tiab] OR "OMNI surgical system"[tiab] OR
  GATT[tiab] OR "gonioscopy assisted transluminal trabeculotomy"[tiab] OR
```

canaloplasty[tiab] OR viscodilation[tiab] OR
"viscodilation of Schlemm canal"[tiab] OR
"trabecular bypass"[tiab] OR "trabecular micro-bypass"[tiab] OR
"Schlemm canal surgery"[tiab] OR
"angle-based surgery"[tiab]
)
AND
(
"Phacoemulsification"[Mesh] OR
phacoemulsification[tiab] OR
phaco[tiab] OR
"cataract surgery"[tiab] OR
"lens extraction"[tiab] OR
"combined surgery"[tiab] OR
"combined with cataract surgery"[tiab] OR
phacotrabecular[tiab]
)
AND
(
"Costs and Cost Analysis"[Mesh] OR
"Cost-Benefit Analysis"[Mesh] OR
"Economics"[Subheading] OR
"cost effectiveness"[tiab] OR "cost-effectiveness"[tiab] OR
"cost utility"[tiab] OR "cost-utility"[tiab] OR
"cost benefit"[tiab] OR "cost-benefit"[tiab] OR
"economic evaluation"[tiab] OR
"health economic*"[tiab] OR
QALY[tiab] OR "quality adjusted life year*"[tiab] OR
ICER[tiab] OR "incremental cost effectiveness ratio"[tiab] OR
ICUR[tiab] OR "incremental cost utility ratio"[tiab] OR
"budget impact"[tiab] OR
"cost analysis"[tiab] OR
"cost minimization"[tiab] OR
"cost savings"[tiab]
)

Embase: PHACO-MIGS + cost-effectiveness
(
'glaucoma'/exp OR

```
  'ocular hypertension'/exp OR
  glaucoma:ti,ab OR
  'primary open angle glaucoma':ti,ab OR poag:ti,ab OR
  'primary angle closure glaucoma':ti,ab OR pacg:ti,ab OR
  'angle closure glaucoma':ti,ab OR acg:ti,ab OR
  'normal tension glaucoma':ti,ab OR ntg:ti,ab OR
  'low tension glaucoma':ti,ab OR
  'secondary glaucoma':ti,ab OR
  'pseudoexfoliation glaucoma':ti,ab OR pxg:ti,ab OR
  'pigmentary glaucoma':ti,ab OR
  'neovascular glaucoma':ti,ab OR
  'uveitic glaucoma':ti,ab OR
  'steroid induced glaucoma':ti,ab OR
  'traumatic glaucoma':ti,ab OR
  'aphakic glaucoma':ti,ab OR
  'pseudophakic glaucoma':ti,ab OR
  'ocular hypertension':ti,ab OR oht:ti,ab
)
AND
(
  'minimally invasive glaucoma surgery'/exp OR
  'glaucoma surgery'/exp OR
  migs:ti,ab OR
  'minimally invasive glaucoma surgery':ti,ab OR
  'microinvasive glaucoma surgery':ti,ab OR
  'minimally invasive glaucoma surgeries':ti,ab OR
  istent:ti,ab OR 'istent inject':ti,ab OR 'istent infinite':ti,ab OR
  hydrus:ti,ab OR 'hydrus microstent':ti,ab OR
  kahook:ti,ab OR 'kahook dual blade':ti,ab OR kdb:ti,ab OR
  trabectome:ti,ab OR 'ab interno trabeculectomy':ti,ab OR
  omni:ti,ab OR 'omni surgical system':ti,ab OR
  gatt:ti,ab OR 'gonioscopy assisted transluminal trabeculotomy':ti,ab OR
  canaloplasty:ti,ab OR viscodilation:ti,ab OR
  'viscodilation of schlemm canal':ti,ab OR
  'trabecular bypass':ti,ab OR 'trabecular micro-bypass':ti,ab OR
  'schlemm canal surgery':ti,ab OR
  'angle-based surgery':ti,ab
)
AND
```

```
(
  'phacoemulsification'/exp OR
  phacoemulsification:ti,ab OR
  phaco:ti,ab OR
  'cataract surgery':ti,ab OR
  'lens extraction':ti,ab OR
  'combined surgery':ti,ab OR
  'combined with cataract surgery':ti,ab OR
  phacotrabecular:ti,ab
)
AND
(
  'cost effectiveness analysis'/exp OR
  'cost utility analysis'/exp OR
  'cost benefit analysis'/exp OR
  'economic evaluation'/exp OR
  'health economics'/exp OR
  'budget impact analysis'/exp OR
  'cost analysis'/exp OR
  'cost effectiveness':ti,ab OR 'cost-effectiveness':ti,ab OR
  'cost utility':ti,ab OR 'cost-utility':ti,ab OR
  'cost benefit':ti,ab OR 'cost-benefit':ti,ab OR
  'economic evaluation':ti,ab OR
  'health economic*':ti,ab OR
  qaly:ti,ab OR 'quality adjusted life year*':ti,ab OR
  icer:ti,ab OR 'incremental cost effectiveness ratio':ti,ab OR
  icur:ti,ab OR 'incremental cost utility ratio':ti,ab OR
  'budget impact':ti,ab OR
  'cost analysis':ti,ab OR
  'cost minimization':ti,ab OR
  'cost savings':ti,ab
)
```

Scopus: PHACO-MIGS + cost-effectiveness

```
TITLE-ABS-KEY
(
  glaucoma OR
  "primary open angle glaucoma" OR POAG OR
  "primary angle closure glaucoma" OR PACG OR
  "angle closure glaucoma" OR ACG OR
  "normal tension glaucoma" OR NTG OR
  "low tension glaucoma" OR
  "secondary glaucoma" OR
  "pseudoexfoliation glaucoma" OR PXG OR
  "pigmentary glaucoma" OR
  "neovascular glaucoma" OR
  "uveitic glaucoma" OR
  "steroid induced glaucoma" OR
  "traumatic glaucoma" OR
  "aphakic glaucoma" OR
  "pseudophakic glaucoma" OR
  "ocular hypertension" OR OHT
)
AND TITLE-ABS-KEY
(
  MIGS OR
  "minimally invasive glaucoma surgery" OR
  "microinvasive glaucoma surgery" OR
  "minimally invasive glaucoma surgeries" OR
  iStent OR "iStent inject" OR "iStent infinite" OR
  Hydrus OR "Hydrus microstent" OR
  Kahook OR "Kahook dual blade" OR KDB OR
  Trabectome OR "ab interno trabeculectomy" OR
  OMNI OR "OMNI surgical system" OR
  GATT OR "gonioscopy assisted transluminal trabeculotomy" OR
  canaloplasty OR viscodilation OR
  "viscodilation of Schlemm canal" OR
  "trabecular bypass" OR "trabecular micro-bypass" OR
  "Schlemm canal surgery" OR
  "angle-based surgery"
)
AND TITLE-ABS-KEY
```

```
(
  phacoemulsification OR
  phaco OR
  "cataract surgery" OR
  "lens extraction" OR
  "combined surgery" OR
  "combined with cataract surgery" OR
  phacotrabecular
)
AND TITLE-ABS-KEY
(
  "cost effectiveness" OR "cost-effectiveness" OR
  "cost utility" OR "cost-utility" OR
  "cost benefit" OR "cost-benefit" OR
  "economic evaluation" OR
  "health economic*" OR
  QALY OR "quality adjusted life year*" OR
  ICER OR "incremental cost effectiveness ratio" OR
  ICUR OR "incremental cost utility ratio" OR
  "budget impact" OR
  "cost analysis" OR
  "cost minimization" OR
  "cost savings"
)
```

3

Supplementary Chart S2

Supplementary Chart S2: Drummond's checklist for the critical appraisal of economic evaluations

1 Was a well-defined question posed in answerable form?
1.1 Did the study examine both costs and effects of the service(s) or programme(s)?
Yes – examine the cost. Cost utility analysis
Effect-efficacy & safety
1.2 Did the study involve a comparison of alternatives?
Yes. Comparing the CS alone
1.3 Was a viewpoint for the analysis stated and was the study placed in any particular decision-making context? yes

2 Was a comprehensive description of the competing alternatives given (*i.e. can you tell who, did what, to whom, where and how often*)?
2.1 Were any important alternatives omitted?
2.2 Was (*Should*) a *do-nothing* alternative (*be*) considered?

3 Was the effectiveness of the programmes or services established?
3.1 Was this done through a randomised controlled clinical trial? If so, did the study protocol reflect what would happen in regular practice?
3.2 Was effectiveness established through an overview of clinical studies (*systematic review/meta-analysis*)?
3.3 Were observational data or assumptions used to establish effectiveness? If so, what are the potential biases in results?

4 Were all the important and relevant costs and consequences for each alternative identified?
4.1 Was the range wide enough for the research question at hand?
4.2 Did it cover all relevant viewpoints? (*possible viewpoints include the community or social viewpoint, and those of patients and third-party payers*)
4.3 Were capital costs, as well as operating costs, included?

5 Were costs and consequences measured accurately in appropriate physical units (*e.g. hours of nursing time, number of physician visits, lost work days, gained life-years*)?
5.1 Were any of the identified items omitted from measurement? If so, does this mean that they carried no weight in the subsequent analysis?
5.2 Were there any special circumstances (*e.g. joint use of resources*) that made measurement difficult? Were these circumstances handled appropriately?

6 Were costs and consequences valued credibly?
6.1 Were the sources of all values clearly identified? (*possible sources include market values, patient preferences and views, policy-makers' views and health professionals' judgements*)
6.2 Were market values employed for changes involving resources gained or depleted?
6.3 Where market values were absent (*e.g. volunteer labour*), or market values did not reflect actual values (*such as clinic space donated at a reduced rate*), were adjustments made to approximate market values?
6.4 Was the valuation of consequences appropriate for the question posed (*i.e. has the appropriate type or types of analysis – cost-effectiveness, cost–benefit, cost–utility – been selected*)?

7 Were costs and consequences adjusted for differential timing?
7.1 Were costs and consequences that occur in the future 'discounted' to their present values?
7.2 Was any justification given for the discount rate used?

8 Was an incremental analysis of costs and consequences of alternatives performed?
8.1 Were the additional (*incremental*) costs generated by one alternative over another compared with the additional effects, benefits, or utilities generated?

9 Was allowance made for uncertainty in the estimates of costs and consequences?
9.1 If data on costs or consequences were stochastic, were appropriate statistical analyses performed?
9.2 If a sensitivity analysis was employed, was justification provided for the ranges of values (*for key study parameters*)?
9.3 Were study results sensitive to changes in the values (*within the assumed range for sensitivity analysis, or within the CI around the ratio of costs to outcomes*)?

10 Did the presentation and discussion of study results include all issues of concern to users?
10.1 Were the conclusions of the analysis based on some overall index or ratio of costs to consequences (*e.g. cost-effectiveness ratio*)? If so, was the index interpreted intelligently or in a mechanistic fashion?
10.2 Were the results compared with those of others who have investigated the same question? If so, were allowances made for potential differences in study methodology?
10.3 Did the study discuss the generalisability (*external validity*) of the results to other settings and patient/client groups?
10.4 Did the study allude to, or take account of, other important factors in the choice or decision under consideration (*e.g. distribution of costs and consequences, or ethical issues*)?
10.5 Did the study discuss issues of implementation, such as the feasibility of adopting the 'preferred' programme given existing financial or other constraints, and whether any freed resources could be redeployed to other worthwhile programmes?
