# Supplementary material for: Cost-Effectiveness of Angle-Based Minimally Invasive Glaucoma Surgery (MIGS): A Systematic Review of Economic Evaluations
Source: Med Sci (Basel). 2026 Oct 12;14(6):664. doi: 10.3390/medsci14060664 (PMC13707156; doi:10.3390/medsci14060664)
Supplement: Supplementary file 1 [file medsci-14-00664-s001.zip › Table S1-PRISMA_2020_checklist_MIGS_CE(R).pdf]

PRISMA 2020 Checklist

Section and Topic	Item #	Checklist item	Location where item is reported
**TITLE**			
Title	1	Identify the report as a systematic review.	Title page, lines 1–2
**ABSTRACT**			
Abstract	2	See the PRISMA 2020 for Abstracts checklist.	Done. Please refer to PRISMA abstract checklist
**INTRODUCTION**			
Rationale	3	Describe the rationale for the review in the context of existing knowledge.	Introduction (Line 162-181)
Objectives	4	Provide an explicit statement of the objective(s) or question(s) the review addresses.	Introduction (Line 183-187)
**METHODS**			
Eligibility criteria	5	Specify the inclusion and exclusion criteria for the review and how studies were grouped for the syntheses.	Methods, Eligibility Criteria, line 209
Information sources	6	Specify all databases, registers, websites, organisations, reference lists and other sources searched or consulted to identify studies. Specify the date when each source was last searched or consulted.	Methods, Search Strategy, line 202
Search strategy	7	Present the full search strategies for all databases, registers and websites, including any filters and limits used.	Methods, Search Strategy, line 202 Supplementary Table 1.
Selection process	8	Specify the methods used to decide whether a study met the inclusion criteria of the review, including how many reviewers screened each record and each report retrieved, whether they worked independently, and if applicable, details of automation tools used in the process.	Methods, line 233: two reviewers, independent title/abstract screening, full-text assessment, masking and disagreement resolution.
Data collection process	9	Specify the methods used to collect data from reports, including how many reviewers collected data from each report, whether they worked independently, any processes for obtaining or confirming data from study investigators, and if applicable, details of automation tools used in the process.	Methods, Data Extraction, lines 239-246: one extractor, independent verification by a second reviewer/
Data items	10a	List and define all outcomes for which data were sought. Specify whether all results that were compatible with each outcome domain in each study were sought (e.g. for all measures, time points, analyses), and if not, the methods used to decide which results to collect.	Methods, Outcomes and Economic Measures, lines 248-255; synthesis classification, lines 273-292
	10b	List and define all other variables for which data were sought (e.g. participant and intervention characteristics, funding sources). Describe any assumptions made about any missing or unclear information.	Methods, Data Extraction, line 245-246
Study risk of bias assessment	11	Specify the methods used to assess risk of bias in the included studies, including details of the tool(s) used, how many reviewers assessed each study and whether they worked independently, and if applicable, details of automation tools used in the process.	Methods, Quality Assessment, lines 294-313
Effect measures	12	Specify for each outcome the effect measure(s) (e.g. risk ratio, mean difference) used in the synthesis or presentation of results.	Methods, Outcomes and Economic Measures, Lines 248-271
Synthesis	13a	Describe the processes used to decide which studies were eligible for each synthesis (e.g. tabulating the study intervention characteristics and	Methods, lines 273-292

Section and Topic	Item #	Checklist item	Location where item is reported
methods		comparing against the planned groups for each synthesis (item #5)).	
	13b	Describe any methods required to prepare the data for presentation or synthesis, such as handling of missing summary statistics, or data conversions.	Methods, Data Preparation and Synthesis: lines 273-292
	13c	Describe any methods used to tabulate or visually display results of individual studies and syntheses.	Methods, Data Preparation and Synthesis: lines 273-292
	13d	Describe any methods used to synthesize results and provide a rationale for the choice(s). If meta-analysis was performed, describe the model(s), method(s) to identify the presence and extent of statistical heterogeneity, and software package(s) used.	NA
	13e	Describe any methods used to explore possible causes of heterogeneity among study results (e.g. subgroup analysis, meta-regression).	NA
	13f	Describe any sensitivity analyses conducted to assess robustness of the synthesized results.	Methods, Data Preparation and Synthesis: lines 289-292
Reporting bias assessment	14	Describe any methods used to assess risk of bias due to missing results in a synthesis (arising from reporting biases).	Line 294-323
Certainty assessment	15	Describe any methods used to assess certainty (or confidence) in the body of evidence for an outcome.	Line 323
**RESULTS**			
Study selection	16a	Describe the results of the search and selection process, from the number of records identified in the search to the number of studies included in the review, ideally using a flow diagram.	Results, Literature Search Results, 342-386
	16b	Cite studies that might appear to meet the criteria, but which were excluded, and explain why they were excluded.	Figure 1 lists exclusion categories and counts
Study characteristics	17	Cite each included study and present its characteristics.	Tables 1–2; Results, Study Characteristics
Risk of bias in studies	18	Present assessments of risk of bias for each included study.	Drummond methodological appraisal: Results, Quality Assessment. CHEERS reporting completeness
Results of individual studies	19	For all outcomes, present, for each study: (a) summary statistics for each group (where appropriate) and (b) an effect estimate and its precision (e.g. confidence/credible interval), ideally using structured tables or plots.	Table 2; Results, Primary Outcomes and comparison-specific subsections
Results of syntheses	20a	For each synthesis, briefly summarise the characteristics and risk of bias among contributing studies.	Results, comparison-specific subsections, Tables 1–3.
	20b	Present results of all statistical syntheses conducted. If meta-analysis was done, present for each the summary estimate and its precision (e.g. confidence/credible interval) and measures of statistical heterogeneity. If comparing groups, describe the direction of the effect.	Descriptive synthesis: Table 3; Figure 2, p. 24; Results, Primary Outcomes and comparison subsections,
	20c	Present results of all investigations of possible causes of heterogeneity among study results.	Descriptive differences are presented in comparison-specific Results.

PRISMA

PRISMA 2020 Checklist

Section and Topic	Item #	Checklist item	Location where item is reported
	20d	Present results of all sensitivity analyses conducted to assess the robustness of the synthesized results.	No review-level sensitivity-analysis results are reported. Table 1 and selected Table 2 remarks describe sensitivity analyses within original economic studies.
Reporting biases	21	Present assessments of risk of bias due to missing results (arising from reporting biases) for each synthesis assessed.	Table 4-6
Certainty of evidence	22	Present assessments of certainty (or confidence) in the body of evidence for each outcome assessed.	Not undertaken: Methods, Quality Assessment, states that formal body-of-evidence certainty assessment was not performed. Accordingly, no outcome-level certainty ratings are presented.
**DISCUSSION**			
Discussion	23a	Provide a general interpretation of the results in the context of other evidence.	Discussion: interpretation in relation to economic and clinical evidence. Reconcile claims of dominance with the comparison-level results before final submission.
	23b	Discuss any limitations of the evidence included in the review.	Discussion: model-based evidence, geographic/generalizability limitations, few head-to-head comparisons and heterogeneous assumptions.
	23c	Discuss any limitations of the review processes used.	Discussion: explain inability to pool; English/full-report restrictions, retrieval/search limitations.
	23d	Discuss implications of the results for practice, policy, and future research.	Discussion; Conclusions and Future Directions.
**OTHER INFORMATION**			
Registration and protocol	24a	Provide registration information for the review, including register name and registration number, or state that the review was not registered.	Methods, Reporting guidelines and Registration: PROSPERO, CRD420261462492.
	24b	Indicate where the review protocol can be accessed, or state that a protocol was not prepared.	Methods, Reporting guidelines and Registration: registered methods are available in PROSPERO; no separate review protocol was prepared.
	24c	Describe and explain any amendments to information provided at registration or in the protocol.	Not reported.
Support	25	Describe sources of financial or non-financial support for the review, and the role of the funders or sponsors in the review.	Funding statement: National Medical Research Council support for an author and the grant body's lack of involvement. Confirm whether any additional financial or non-financial support specifically supported the review.
Competing interests	26	Declare any competing interests of review authors.	Conflicts of Interest statement: none declared.
Availability of data, code and other materials	27	Report which of the following are publicly available and where they can be found: template data collection forms; data extracted from included studies; data used for all analyses; analytic code; any other materials used in the review.	Data Availability Statement: no new data were created or analyzed and sharing is not applicable

From: Page MJ, McKenzie JE, Bossuyt PM, Boutron I, Hoffmann TC, Mulrow CD, et al. The PRISMA 2020 statement: an updated guideline for reporting systematic reviews. BMJ 2021;372:n71. doi: 10.1136/bmj.n71.
This work is licensed under CC BY 4.0. To view a copy of this license, visit https://creativecommons.org/licenses/by/4.0/
